# Pre‐Conditioned Bone Marrow Mesenchymal Stromal Cell‐Derived Secretome Exerts an Anti‐Inflammatory Effect on Degenerative Nucleus Pulposus Cells In Vitro

**DOI:** 10.1002/jsp2.70199

**Published:** 2026-06-20

**Authors:** Veronica Tilotta, Gianluca Vadalà, Luca Ambrosio, Giuseppina Di Giacomo, Claudia Cicione, Fabrizio Russo, Rocco Papalia, Vincenzo Denaro

**Affiliations:** ^1^ Laboratory for Regenerative Orthopaedics, Operative Research Unit of Orthopaedic and Trauma Surgery Fondazione Policlinico Universitario Campus Bio‐Medico Rome Italy; ^2^ Research Unit of Orthopaedic and Trauma Surgery, Departmental Faculty of Medicine and Surgery Università Campus Bio‐Medico di Roma Rome Italy

**Keywords:** disc degeneration, low back pain, mesenchymal stromal cells, regeneration, secretome, spine

## Abstract

**Background:**

Intervertebral disc degeneration (IDD) is frequently associated with chronic low back pain (LBP), which contributes significantly to disability, psychological distress, and reduced work capacity. Mesenchymal stromal cell (MSC)‐based treatments may offer a promising, less invasive alternative to conventional treatments for IDD. The MSC secretome has shown regenerative potential through paracrine and anti‐inflammatory mechanisms. In this study, we investigated the effects of the secretome isolated from interleukin‐1β (IL‐1β)‐preconditioned bone marrow‐derived MSCs (BM‐MSCs) on human nucleus pulposus cells (hNPCs) adopting a 3D in vitro culture model.

**Methods:**

The secretome (BM‐MSCsec) was collected from BM‐MSCs preconditioned with 10 ng/mL IL‐1β. hNPCs were isolated, expanded, encapsulated in alginate beads, and stimulated with IL‐1β to mimic a pro‐inflammatory environment. These cells were then treated with either standard culture media (control group), IL‐1β alone, BM‐MSCsec, or IL‐1β + BM‐MSCsec. We evaluated cell proliferation and viability via flow cytometry, nitrite and reactive oxygen species (ROS) levels using the H2DCFDA assay, glycosaminoglycan (GAG) content with the 1,9‐dimethylmethylene blue assay, gene expression of extracellular matrix (ECM) components and inflammatory markers via qPCR, and cell senescence through Western blot and β‐galactosidase staining.

**Results:**

IL‐1β stimulation increased hNPC proliferation, nitrite release, ROS production, catabolic and inflammatory gene expression, and cell senescence. Treatment with BM‐MSCsec attenuated these effects, restoring proliferation to baseline levels, significantly reducing ROS and nitrite accumulation. BM‐MSCsec also upregulated anabolic genes (*ACAN*, *COL2A1*, *SOX9*, *TIMP‐1/3*), enhanced GAG production, and downregulated *IL‐1β*, *IL‐6*, *IL‐8*, *NOS2*, *MMP‐1*, and *MMP‐13* expression. Furthermore, senescence was markedly reduced in BM‐MSCsec–treated hNPCs, with decreased β‐galactosidase activity and lower p16 and p21 expression.

**Conclusions:**

Our findings support the potential of BM‐MSCsec as a cell‐free therapeutic strategy for IDD. The IL‐1β‐preconditioned secretome reduced hNPC death and senescence, mitigated inflammation and oxidative stress, and promoted ECM preservation, highlighting its potential to counteract key processes driving IDD.

## Graphical abstract

IL‐1β‐preconditioned BM‐MSC‐derived secretome counteracts inflammation‐driven degeneration in hNPCs. BM‐MSCs were preconditioned with IL‐1β (10 ng/mL) for 24 h, and the resulting secretome (BM‐MSCsec) was collected. Degenerative hNPCs cultured in a 3D alginate bead model were exposed to IL‐1β to reproduce an inflammatory degenerative microenvironment and subsequently treated with BM‐MSCsec. IL‐1β stimulation induced a degenerative phenotype characterized by increased inflammatory mediators, oxidative stress, matrix degradation, and cell senescence, together with reduced GAG content. Treatment with BM‐MSCsec attenuated inflammation, ROS production, MMP‐mediated catabolism, and senescence markers, while promoting anabolic ECM‐related gene expression and GAG production. These findings support IL‐1β‐preconditioned BM‐MSCsec as a promising cell‐free therapeutic approach for intervertebral disc degeneration. Abbreviations: BM‐MSC = bone marrow‐derived mesenchymal stromal cell; BM‐MSCsec = bone marrow‐derived mesenchymal stromal cell‐derived secretome; ECM = extracellular matrix; GAG = glycosaminoglycan; hNPC = human nucleus pulposus cell; IL = interleukin; MMP = matrix metalloproteinase; ROS = reactive oxygen species.

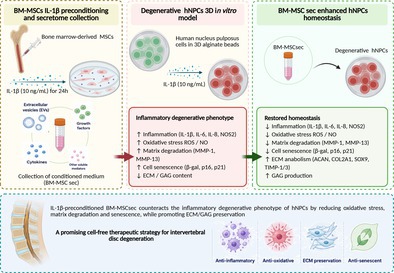

## 1 Introduction

Chronic low back pain (LBP) is a highly prevalent and progressively debilitating condition, particularly among the elderly, leading to persistent pain and disability [1]. Among its various etiologies, intervertebral disc degeneration (IDD) emerges as a key contributor to the chronicity of LBP, accounting for a substantial healthcare burden, including an estimated annual loss of approximately 149 million workdays worldwide [2].

Despite the high prevalence and societal impact of LBP, current treatment options, ranging from conservative strategies (e.g., analgesic drugs, physiotherapy) to surgical interventions, often fail to provide sustained pain relief or restore normal disc function [3]. While the intradiscal injection of mesenchymal stromal cells (MSCs) has shown promise in fostering disc repair, challenges such as poor post‐transplant cell viability, undesired differentiation, and donor‐to‐donor variability limit its widespread application [4, 5]. Consequently, cell‐free therapies have been gaining increasing traction, particularly those leveraging the MSC‐derived secretome, a complex mix of extracellular vesicles (EVs), chemokines, growth factors, and cytokines mediating immunomodulatory and regenerative effects. This secretome can influence the disc microenvironment by modulating endogenous cell behavior and enhancing cell–cell and cell–matrix interactions [6, 7]. Its therapeutic potential has been demonstrated in various disease models, including myocardial infarction, where the MSC secretome reduced cardiomyocyte apoptosis and promoted angiogenesis [8, 9], and nerve injury, supporting neural regeneration and tissue repair [10].

Recent evidence has shown that preconditioning MSCs through physical, chemical, or biological stimuli can enhance their paracrine activity and therapeutic efficacy [11]. Hypoxic preconditioning has been demonstrated to stimulate the production of proangiogenic factors like vascular endothelial growth factor (VEGF), upregulate the secretion of anti‐inflammatory cytokines, and improve MSC survival and engraftment post‐transplantation [12]. On the other hand, pre‐treating MSCs with inflammatory cytokines like tumor necrosis factor (TNF)‐α, interferon (IFN)‐γ, and interleukin (IL)‐1β has been shown to increase the secretion of immunosuppressive and anti‐inflammatory factors [13]. Similarly, pharmacological agents and the application of mechanical forces (e.g., stretching or compression) have been demonstrated to boost MSC regenerative properties, improve cell differentiation toward specific lineages, and enhance the secretion of matrix components and growth factors [14]. Moreover, 3D culture systems may also represent a type of preconditioning by mimicking the physiological cell niche in vitro [15].

In this study, we investigated the paracrine and anti‐inflammatory effects of the secretome isolated from IL‐1β‐preconditioned bone marrow‐derived MSCs (BM‐MSCs) on human nucleus pulposus cells (hNPCs) adopting a three‐dimensional in vitro model. Our goal was to explore the regenerative and protective capacity of this enhanced secretome to counteract the degenerative and inflammatory processes associated with IDD.

## 2 Materials and Methods

**FIGURE 1:**
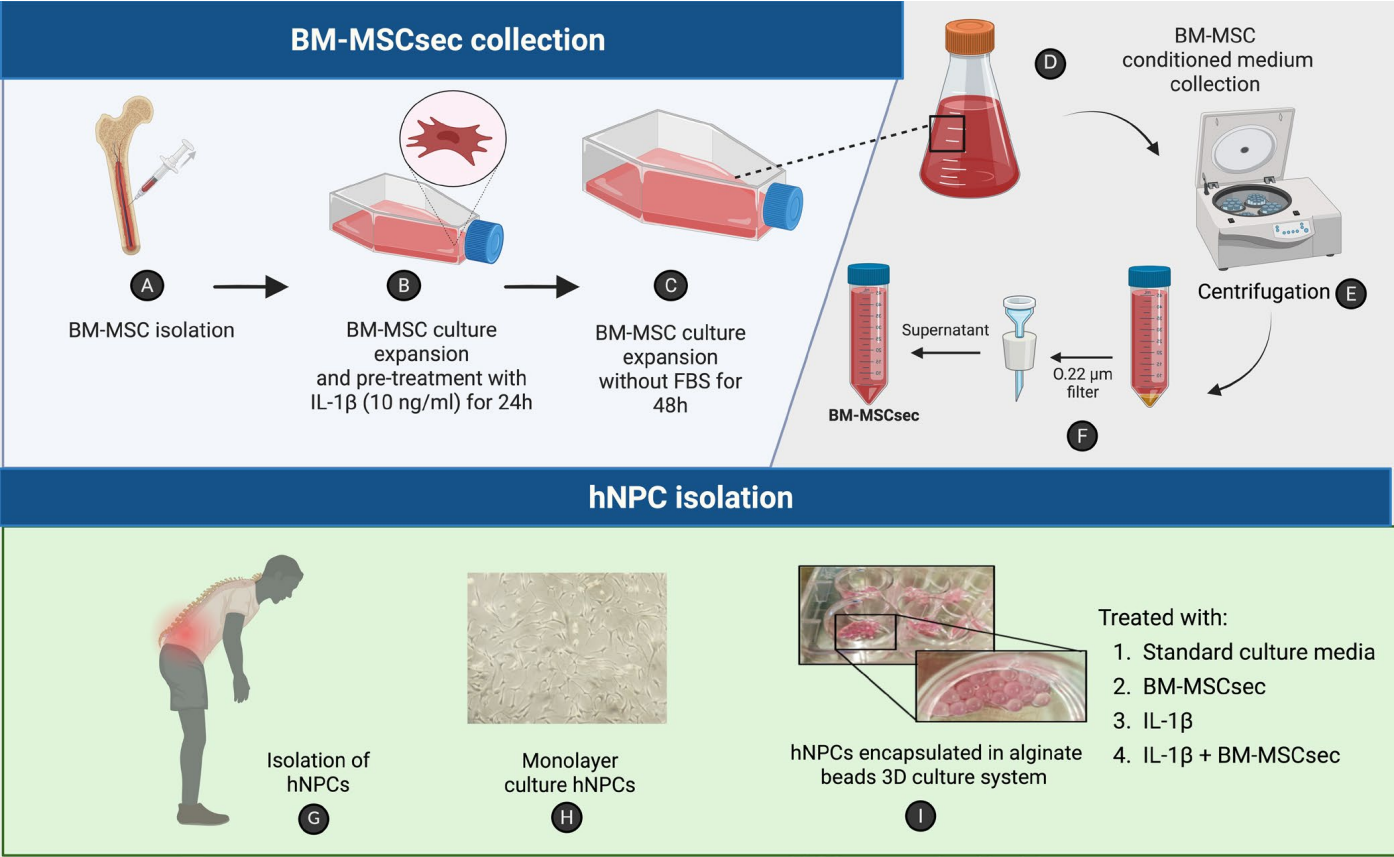
Schematic overview of the experimental workflow. Human BM‐MSCs were obtained from healthy donors via iliac crest aspiration (A), then cultured using a standard medium supplemented with 10 ng/mL IL‐1β (B). After expansion for 48 h in a standard medium without FBS (C), conditioned media were collected (D), processed for secretome isolation (E), filtered (F), and stored until use. Degenerative hNPCs were isolated from patients undergoing discectomy (G), cultured in monolayer (H), then encapsulated in a 3D alginate bead system (I) and treated with one of the following conditions: standard culture medium, BM‐MSCsec, IL‐1β (10 ng/mL), or IL‐1β (10 ng/mL for 24 h) followed by co‐incubation with BM‐MSC sec. Created with BioRender.com. Abbreviations: BM‐MSC = bone marrow–derived mesenchymal stromal cells; BM‐MSCsec = bone marrow–derived mesenchymal stromal cell secretome; FBS = fetal bovine serum; hNPCs = human nucleus pulposus cells; IL = interleukin.

The study was performed in accordance with the Declaration of Helsinki, and the study protocol was approved by the Ethics Committee of Università Campus Bio‐Medico di Roma (n. 09/15 PAR ComEt CBM). hNPCs and BM‐MSCs were harvested from surgical waste specimens of human donors after collection of written informed consent. Experimental procedures are summarized in Figure 1.

### 2.1 Isolation and Culture of BM‐MSC

Human BM‐MSCs were isolated from 3 healthy donors (M:F = 2:1; mean age = 45.0 ± 11.0 years old, no comorbidities) as previously described [6]. Briefly, bone marrow was harvested from the posterior superior iliac spine with 20 mL‐syringes containing 500 IU of preservative‐free heparin (PharmaTex Italia, Milan, Italy). Cells were expanded in Dulbecco's Modification of Eagle's Medium (DMEM; Corning, Corning, NY, USA) + 1% penicillin/streptomycin (P/S; Sigma, St. Louis, MO, USA) + 20% heat‐inactivated fetal bovine serum (FBS) at 37°C with 5% humidified CO_2_. Non‐adherent cells were removed by culture media change at 1 day of incubation, whereas adherent cells were expanded until 70%–80% of confluency up to the sixth passage (P6). BM‐MSCs were characterized according to the International Society for Cell and Gene Therapy (ISCT) criteria [16] in terms of surface marker expression and multilineage differentiation, as shown in our previous study [6].

### 2.2 IL‐1β Preconditioning and Secretome Collection

BM‐MSCs at 70%–80% of confluency were rinsed twice with phosphate‐buffered saline (PBS) and incubated with the culture medium supplemented with 10 ng/mL IL‐1β (Peprotech, Thermo Fisher Scientific, Waltham, MA, USA) for 24 h. After incubation with IL‐1β, BM‐MSCs were thoroughly rinsed with PBS and cultured in DMEM supplemented with 1% P/S and without FBS. Following 48 h of incubation, the BM‐MSC‐derived secretome (BM‐MSCsec) was collected and processed according to Ragni et al. [17], with minor changes. Briefly, the conditioned medium was sequentially centrifuged at 4°C to remove cellular debris (10 min at 300 g, 10 min at 2000 g, 10 min at 4000 g). The supernatant was then filtered through a 0.22‐μm membrane to ensure removal of residual particles. The BM‐MSCsec was stored at −80°C until further use.

### 2.3 hNPC Isolation and Culture

hNPCs were isolated from the intervertebral disc (IVD) specimens of patients (*n* = 5, mean age = 57 ± 17 years old) undergoing discectomy for lumbar disc herniation as previously described [18]. Demographics, diagnosis, operated level(s), modified Pfirrmann grade of harvested IVDs are shown in Table 1. Specimens were rinsed in PBS, and the nucleus pulposus (NP) tissue was carefully dissected from the annulus fibrosus (AF) and cartilaginous endplates. The NP tissue was minced and digested overnight at 37°C in DMEM +1% P/S + 5% FBS + 0.01% collagenase type II (Worthington Biochemical, Lakewood, NJ, USA). The obtained solution was filtered through a 70‐μm cell strainer, washed, and resuspended in DMEM +10% FBS + 1% P/S + 50 μg/mL L‐ascorbic acid (Sigma) at 37°C in 5% CO_2_ and 21% O_2_, according to the Orthopedic Research Society (ORS) Spine Section guidelines [19]. Culture media were changed two times per week, and cells were expanded until reaching 90% of confluency. Adherent hNPCs at P1 were detached using trypsin–EDTA (Corning), rinsed with PBS, and centrifuged at 1500 rpm for 5 min. Subsequently, cells were resuspended in 1.2% low‐viscosity sodium alginate (Pronova Biopolymer, Drammen, Norway) at a concentration of 4 × 10^6^ cells/mL. The suspension was extruded using a 21‐gauge needle into a 102 mmol/L calcium chloride solution to prepare semisolid beads. Following 10 min of polymerization, beads were rinsed and incubated in DMEM +10% FBS + 1% P/S for 10 days, with media changes twice per week. hNPCs were then divided into four groups: (1) culture media alone (control group); (2) BM‐MSCsec diluted 1:1 in culture media; (3) 10 ng/mL IL‐1β; (4) pre‐treatment with 10 ng/mL IL‐1β for 24 h (before the experimental timeline, i.e., day −1) followed by co‐treatment with 10 ng/mL IL‐1β and 1:1 culture media:BM‐MSCsec.

**TABLE 1:** Summary of characteristics of the donor IVD samples.

Patient ID	Age (years)	Sex	BMI (kg/m^2^)	Level(s)	mPfirrmann grade	Comorbidities
1	38	M	29.3	L5‐S1	4/8	—
2	75	F	26.4	L4‐L5	6/8	HTN, T2DM, CKD, dyslipidemia, hypothyroidism
3	45	M	23.3	L4‐L5	6/8	—
4	44	M	29.9	L4‐L5	5/8	HTN
5	55	F	21.9	L5‐S1	7/8	—
6	67	M	26.0	L5‐S1	7/8	HTN, dyslipidemia, BPH
7	75	M	30.4	L2‐L3	6/8	—
8	88	M	31.4	L2‐L3	6/8	HTN, dyslipidemia
9	52	F	21.5	L4‐L5, L5‐S1	5/8	HTN, dyslipidemia
10	58	F	25.8	L4‐L5	6/8	—
11	56	F	23.3	L4‐L5	6/8	Depression
12	67	F	19.9	L3‐L4	5/8	—

*Note:* Age, sex, BMI, operated level(s), and modified Pfirrmann grading are listed.

Abbreviations: BMI = body mass index; BPH = benign prostatic hyperplasia; CKD = chronic kidney disease; HTN = hypertension; IVD = intervertebral disc; T2DM = type 2 diabetes mellitus.

### 2.4 Cell Viability and Proliferation

To assess the effect of BM‐MSCsec on hNPCs viability, cell membrane integrity was evaluated 24 h post‐treatment using Fixable Viability Dye eFluor780 (Affymetrix eBioscience, Thermo Fisher Scientific). Flow cytometry was performed following the gating strategy described in our previous work [20, 21] to assess the number of viable cells at 1, 3, and 7 days. Results were expressed as events/μL and normalized as percent variation compared to the controls. For analysis, alginate beads were dissolved in a solution containing 55 mmol/L sodium citrate, 30 mmol/L EDTA, and 0.15 M NaCl (pH 6.8). Subsequently, cell count and viability were measured with a CytoFLEX flow cytometer (Beckman Coulter) and analyzed with CytExpert Software (version 2.1).

### 2.5 Assessment of hNPC Nitrite Concentration

Nitrite concentration in the culture supernatant was assessed via the Griess reaction as a proxy of nitric oxide (NO) production. At specific timepoints (1, 3, and 7 days) following treatment, supernatant samples were collected and incubated with 20 μL of Griess reagent (Invitrogen, Carlsbad, CA, USA) for 30 min in the dark, as per the manufacturer's protocol. Absorbance was measured at 546 nm with an Infinite M200 PRO microplate reader (Tecan Life Sciences, Männedorf, Switzerland). Nitrite levels were measured utilizing a sodium nitrite standard curve, and results were expressed as fold changes compared to the untreated control group.

### 2.6 Level of Intracellular Reactive Oxygen Species

hNPCs were seeded in a black 96‐well plate, incubated with 200 μM H_2_O_2_ for 2 h to stimulate reactive oxygen species (ROS) production, and subsequently with 20 μM of 2′,7′‐dichlorofluorescein diacetate (H_2_DCFDA; Merck Millipore, Burlington, MA, USA) at 37°C in the dark for 45 min [22]. After incubation and dye removal, cells were washed with PBS and then cultured for an additional 3 h in HBSS (BioWest L0607; without calcium, magnesium, or phenol red, with sodium bicarbonate). H_2_DCFDA is oxidized by intracellular ROS to 2′,7′‐dichlorofluorescein (DCF), which was quantified with an Infinite M200 PRO microplate reader (Tecan Life Sciences) at excitation/emission wavelengths of 485/535 nm. Fluorescence intensity was measured at 0, 15, 30, 60, 120, and 180 min. In parallel, to visualize DCF fluorescence, cell nuclei were counterstained with 4′,6‐diamidino‐2‐phenylindole (DAPI; SERVA Serving Scientists, Heidelberg, Germany) and imaged utilizing a confocal laser scanning microscope (LSM700, Carl Zeiss, Jena, Germany).

### 2.7 Glycosaminoglycan Content

After 7 days of treatment, after alginate bead dissolution, hNPCs were digested at 65°C in 100 μL papain (0.25 mg/mL in 50 mM phosphate buffer, pH 1.5, with 5 mM cysteine hydrochloride and 5 mM EDTA; Sigma) overnight under gentle shaking to evaluate glycosaminoglycan (GAG) content. GAGs were quantified using the 1,9‐dimethylmethylene blue assay (DMMB; Polysciences, Warrington, PA, USA), with chondroitin sulfate (Sigma) as the standard. Absorbance was measured at 540 nm using an Infinite M200 PRO microplate reader (Tecan Life Sciences). DNA content was assessed using the PicoGreen assay (Invitrogen), based on a standard curve of known DNA concentrations. Fluorescence was measured at excitation/emission wavelengths of 488/520 nm with the same microplate reader. GAG levels were then normalized to DNA content.

### 2.8 RNA Extraction and Gene Expression Analysis

At 3 or 7 days of treatment, alginate beads were digested, and RNA was isolated from cell extracts utilizing the TRIzol reagent (Invitrogen). cDNA was synthesized with the High‐Capacity cDNA Reverse Transcription kit (Applied Biosystems, Foster City, CA, USA) as per manufacturer's indications. mRNA levels were measured via qRT‐PCR with TaqMan Gene Expression Assays and TaqMan Universal Master Mix II (Thermo Fisher Scientific) using the UNG‐Real Time PCR System Instrument 7900HT FAST according to manufacturer's instructions. Investigated genes included aggrecan (*ACAN*; *n* = 6, Hs0153936), collagen 2A1 (*COL2A1*; *n* = 3 Hs00264051), SRY‐Box Transcription Factor‐9 (*SOX‐9*; *n* = 4, Hs01001343), matrix metalloproteinase‐1 (*MMP‐1*; *n* = 3, Hs00899658), *MMP13* (*n* = 5, Hs00233992), a disintegrin and metalloproteinase with thrombospondin motifs‐5 (*ADAMTS‐5*; *n* = 3, Hs00199841), tissue inhibitor of metalloproteinase‐1 (*TIMP‐1*; *n* = 6, Hs01092512), *TIMP‐3* (*n* = 5, Hs00165949), interleukin‐1 β (*IL‐1* β; *n* = 5, Hs00174097), *IL‐6* (*n* = 4, Hs00174131), *IL‐8* ([*CXCL8*]; *n* = 4, Hs00174103), nitric oxide synthase −2 (*NOS‐2*; *n* = 4, Hs01075529). Glyceraldehyde‐3‐phosphate dehydrogenase (*GAPDH*, Hs03929097) and *actin* (Hs4440040) were utilized as housekeeping genes. Gene expression level was normalized to GAPDH and actin expression and calculated according to the 2^‐ΔΔCt^ method. Gene expression was shown as fold changes compared to the control group.

### 2.9 Cell Senescence

Senescence‐associated β‐galactosidase (SA‐β‐gal) staining (Meridian Bioscience, Cincinnati, OH, USA) was performed to evaluate hNPC senescence. hNPCs were seeded in 24‐well plates (1.5 × 10^4^ cells/well) and treated for 7 days as described above. Cells were rinsed with PBS, fixed, and incubated overnight at 37°C with the SA‐β‐gal staining solution. Following incubation, cells were observed using a phase‐contrast microscope, and images were captured from nine random fields per well at 20× magnification. The percentage of SA‐β‐gal‐positive cells was quantified in a blinded manner by calculating the ratio of stained (senescent) cells to the total number of cells. Results were expressed as percent changes compared to the controls.

### 2.10 Western Blot Analysis

After 7 days of treatment, proteins were isolated from hNPC lysates utilizing the radioimmunoprecipitation assay (RIPA) buffer (Sigma) for 30 min on ice, cleared by centrifugation at 12000 g for 30 min at 4°C, and measured with the detergent compatible (DC) protein assay kit (Bio‐Rad, Hercules, CA, USA). Each sample was loaded on 10% SDS‐PAGE gels, transferred to nitrocellulose membranes using the Trans‐Blot Turbo Transfer System (Bio‐Rad), and incubated with a blocking buffer (TBST 1X with 5% non‐fat dry milk) for 1 h. Membranes were then incubated overnight with a primary antibody shaking at 4°C in TBST 1X + 1% non‐fat dry milk. Anti‐p21 (bs‐10129R, rabbit, 1:1000, Bioss Antibodies, Woburn, MA, USA), anti‐CDKN2A/p16INK4a (A32333, rabbit, 1:1000, ABclonal, Wouburn, MA, USA), and anti‐GAPDH (rabbit, 1:1000, Cell Signaling, Danvers, MA, USA) primary antibodies were used. Anti‐mouse and anti‐rabbit HRP‐conjugated antibodies (1:10000, Abcam, Cambridge, UK) were utilized, and chemiluminescence signals were detected with ChemiDoc (Bio‐Rad) and Quantity One software (Bio‐Rad) for densitometric analyses of the different bands.

### 2.11 Statistical Analysis

Continuous variables are expressed as means ± standard deviations (SD), while categorical variables were reported as absolute frequencies and percentages. Data distribution was assessed using the Wilk–Shapiro test. Statistical analysis was performed with one‐way or two‐way analysis of variance (ANOVA) with Dunnett's or Tukey's post‐tests for multiple comparisons wherever applicable, setting the IL‐1β group as a reference. Specific details on the tests utilized for each analysis are provided in the corresponding figure caption. A *p*‐value < 0.05 was considered statistically significant. All analyses were performed with Prism 10 (GraphPad, San Diego, CA, USA). Within each experiment, 3 technical replicates per donor were utilized.

## 3 Results

### 3.1 BM‐MSC Characterization

**FIGURE 2:**
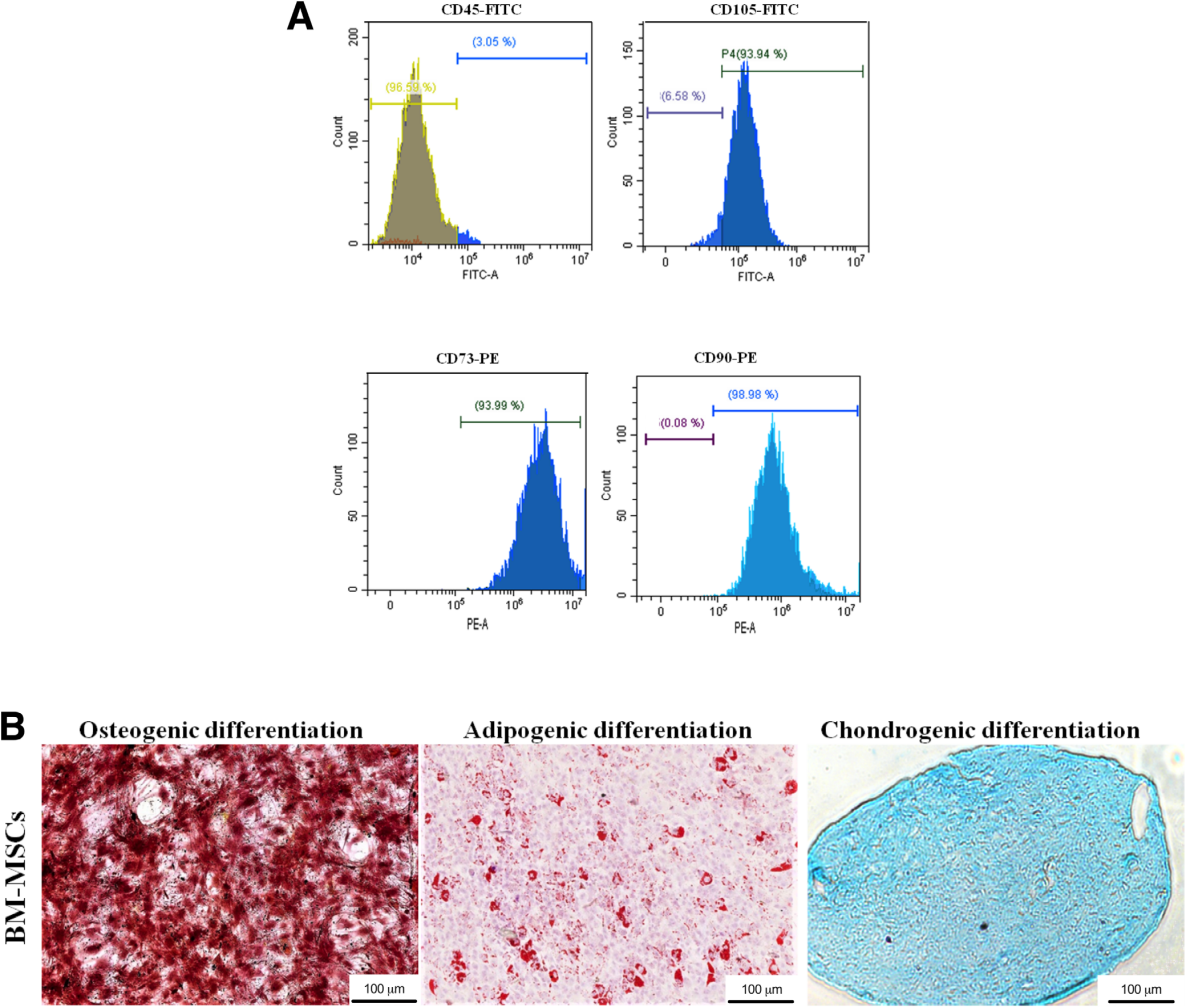
Characterization of BM‐MSCs. (A) Flow cytometry was used to analyze the immunophenotype of BM‐MSCs, confirming positive expression of MSC surface markers CD105, CD73, and CD90, and negative expression of CD45. (B) Multipotency was assessed through lineage‐specific differentiation: Osteogenic, adipogenic, and chondrogenic differentiation were confirmed by Alizarin Red, Oil Red O, and Alcian Blue staining, respectively (magnification: ×20; scale bars: 100 μm). Representative images are shown. Reproduced with permission from Tilotta et al. [6]. Abbreviations: BM‐MSC = bone marrow–derived mesenchymal stromal cells; MSC = mesenchymal stromal cells.

Flow cytometry analysis (Figure 2A) confirmed that BM‐MSCs were positive for the surface markers CD73 (95.9% ± 1.2%), CD90 (98.1% ± 1.0%), and CD105 (96.3% ± 2.4%) and negative for the hematopoietic marker CD45 (1.6% ± 1.3%). Their multipotent potential was confirmed by successful differentiation into chondrogenic, osteogenic, and adipogenic lineages under specific induction conditions (Figure 2B). Adipogenic differentiation was characterized by the formation of lipid droplets visualized with Oil Red O staining, osteogenic differentiation by calcium deposits stained red with Alizarin Red, and chondrogenic differentiation by the detection of cartilaginous ECM using Alcian Blue staining.

### 3.2 BM‐MSCsec Did Not Affect hNPC Viability and Proliferation

**FIGURE 3:**
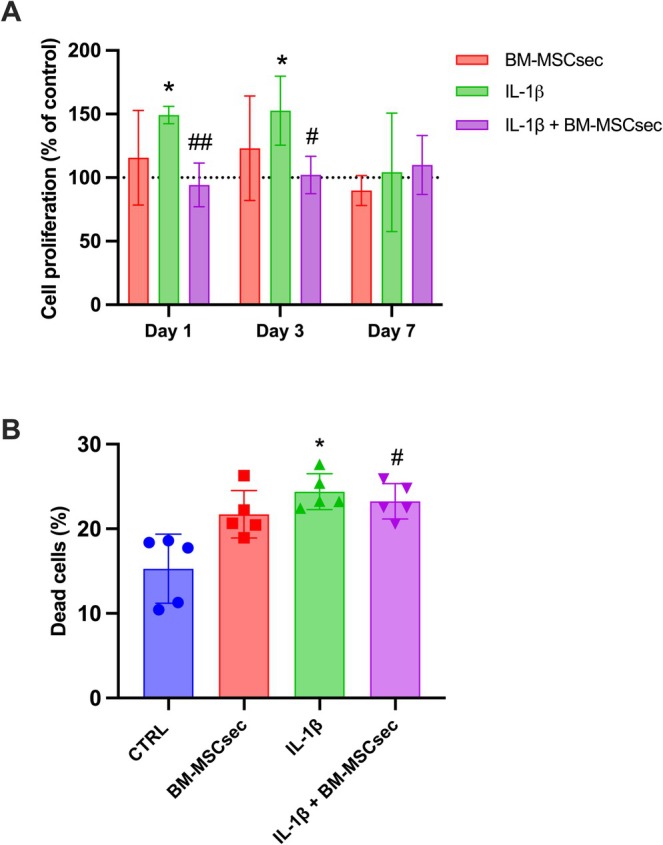
BM‐MSC‐sec modulated hNPC proliferation and viability. (A) Cell number significantly increased following IL‐1β treatment at both 1 and 3 days compared to the control group (*n* = 4). Treatment with BM‐MSCsec alone also led to a modest increase in cell count relative to controls. However, in hNPCs co‐treated with IL‐1β and BM‐MSCsec, cell proliferation was lower than in the IL‐1β–only group, indicating a modulatory effect of the secretome on IL‐1β–induced proliferative activity. Within each timepoint, data were analyzed using unpaired t tests with a false discovery rate approach. Results are expressed as percent variation compared to the controls. The dashed line indicates the reference value (*y* = 100), corresponding to the baseline for percent change normalization. (B) IL‐1β significantly increased hNPC death, while the addition of BM‐MSCsec slightly reduced cell mortality (*n* = 5). Data were analyzed through one‐way ANOVA with Dunnett's multiple comparisons test where each group was compared with IL‐1β. *p < 0.05 compared to the control group; ^#^ *p* < 0.05, ^##^ *p* < 0.01 compared to the IL‐1β group. Abbreviations: BM‐MSCsec = bone marrow–derived mesenchymal stromal cell secretome; hNPC = human nucleus pulposus cell; IL = interleukin.

Flow cytometry was used to evaluate cell proliferation in hNPCs following treatment with BM‐MSCsec, with or without IL‐1β, or with IL‐1β alone (Figure 3A). At days 1 and 3, hNPCs (*n* = 4) treated with IL‐1β alone exhibited a significant increase in cell content compared to controls (149.3% ± 6.8%, *p* = 0.0197; 152.6% ± 27.1%, *p* = 0.0122). On the other hand, co‐treatment with BM‐MSCsec + IL‐1β significantly reduced cell numbers at days 1 and 3 (94.3% ± 17.1%, *p* = 0.0085; 102.1% ± 14.7%, *p* = 0.0166) relative to IL‐1β alone. Treatment with BM‐MSCsec alone did not produce significant changes in cell content at any timepoint. Regarding cell viability (*n* = 5), IL‐1β treatment significantly increased the proportion of dead cells (24.4% ± 2.1%) compared to the control group (15.3% ± 4.0%, *p* = 0.0228). However, co‐treatment with BM‐MSCsec slightly reduced cell death in hNPCs treated with IL‐1β only (23.3% ± 2.1%, *p* = 0.0322; Figure 3B).

### 3.3 BM‐MSCsec Attenuated Nitrite‐Induced Oxidative Stress and Intercellular ROS

**FIGURE 4:**
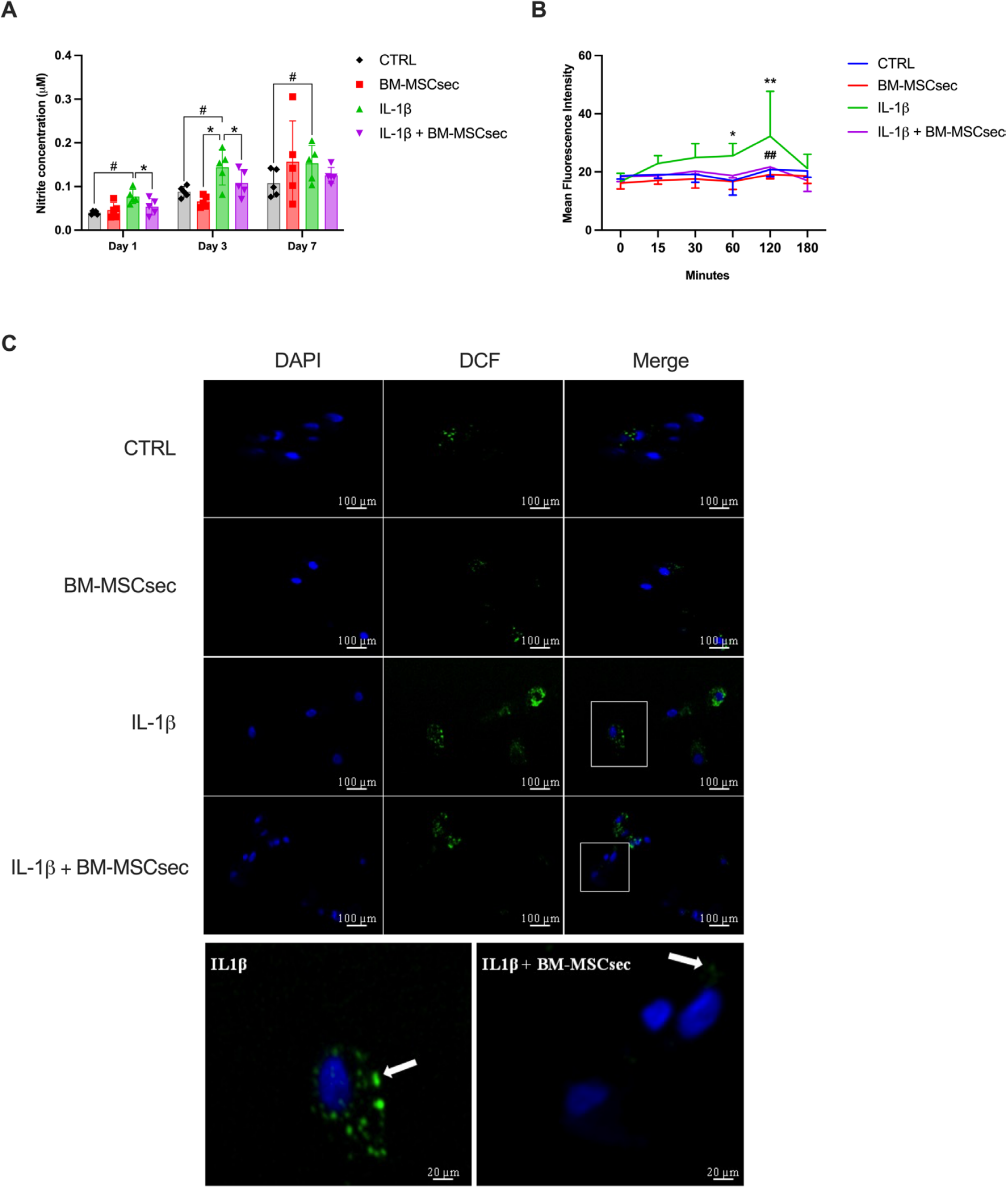
BM‐MSCsec attenuated oxidative stress in hNPCs. (A) BM‐MSCsec and IL‐1β + BM‐MSCsec significantly reduced NO levels at day 1 and day 3 compared to the IL‐1β group (*n* = 5). At each timepoint, data were analyzed through one‐way ANOVA with Dunnett's multiple comparisons test where each group was compared with IL‐1β. *p < 0.05, **p < 0.01, ***p < 0.001. (B) The H_2_DCF‐DA assay showed significantly higher ROS levels in hNPCs treated with IL‐1β alone, with the IL‐1β + BM‐MSCsec showing markedly lower ROS levels (*n* = 3). Data were analyzed through two‐way ANOVA with Dunnett's multiple comparisons test where each group was compared with IL‐1β. *p < 0.05, **p < 0.01, ***p < 0.001 compared to the control group; ^#^ *p* < 0.05, ^##^ *p* < 0.01 compared to the IL‐1β group. (C) Confocal laser scanning microscopy representative images showing lower intracellular ROS localization in hNPCs co‐treated with IL‐1β and BM‐MSCsec. hNPC nuclei were stained with DAPI (blue); DCF probe (green). 20 × magnification. Scale bars = 100 μm. Abbreviations: BM‐MSCsec = bone marrow–derived mesenchymal stromal cell secretome; DCF = 2′,7′‐dichlorofluorescein; H_2_DCFDA = 2′,7′‐dichlorofluorescein diacetate; hNPCs = human nucleus pulposus cells; IL = interleukin; NO = nitric oxide; ROS = reactive oxygen species.

Nitrite release into the supernatant by hNPCs (*n* = 5) was assessed at days 1, 3, and 7 using the Griess reaction (Figure 4A). Notably, NO levels were significantly reduced in the group treated with IL‐1β + BM‐MSCsec compared to the group treated with IL‐1β alone, both at day 1 (0.054 ± 0.019 vs. 0.077 ± 0.016 μM, *p* = 0.0303) and day 3 (0.108 ± 0.030 vs. 0.144 ± 0.040 μM, *p* = 0.0329). In contrast, treatment with IL‐1β alone caused a significant increase in nitrite release at all time points versus the control (day 1: 0.077 ± 0.016 vs. 0.040 ± 0.003 μM, *p* = 0.0192; day 3: 0.144 ± 0.040 vs. 0.088 ± 0.012 μM, *p* = 0.0439; day 7: 0.153 ± 0.041 vs. 0.107 ± 0.031 μM, *p* = 0.0439, *p* = 0.0352). ROS production was evaluated using the H_2_DCF‐DA assay (*n* = 3), which detects intracellular H_2_O_2_‐induced ROS levels. A significant peak in DCF fluorescence was observed at 60 and 120 min in IL‐1β‐treated cells compared to controls (25.50 ± 4.27 vs. 17.00 ± 5.00 and 32.28 ± 15.38 vs. 20.83 ± 2.36; *p* = 0.0453 and *p* = 0.0048, respectively). This increment in ROS was markedly reduced after 120 min by the combination of IL‐1β and BM‐MSCsec compared to IL‐1β group (21.72 ± 3.71 vs. 32.28 ± 15.38; *p* = 0.0099) (Figure 4B). These findings were further supported by representative images captured using confocal laser scanning microscopy (Figure 4C).

### 3.4 BM‐MSCsec Enhanced GAG Content and Regulated ECM Marker Gene Expression

**FIGURE 5:**
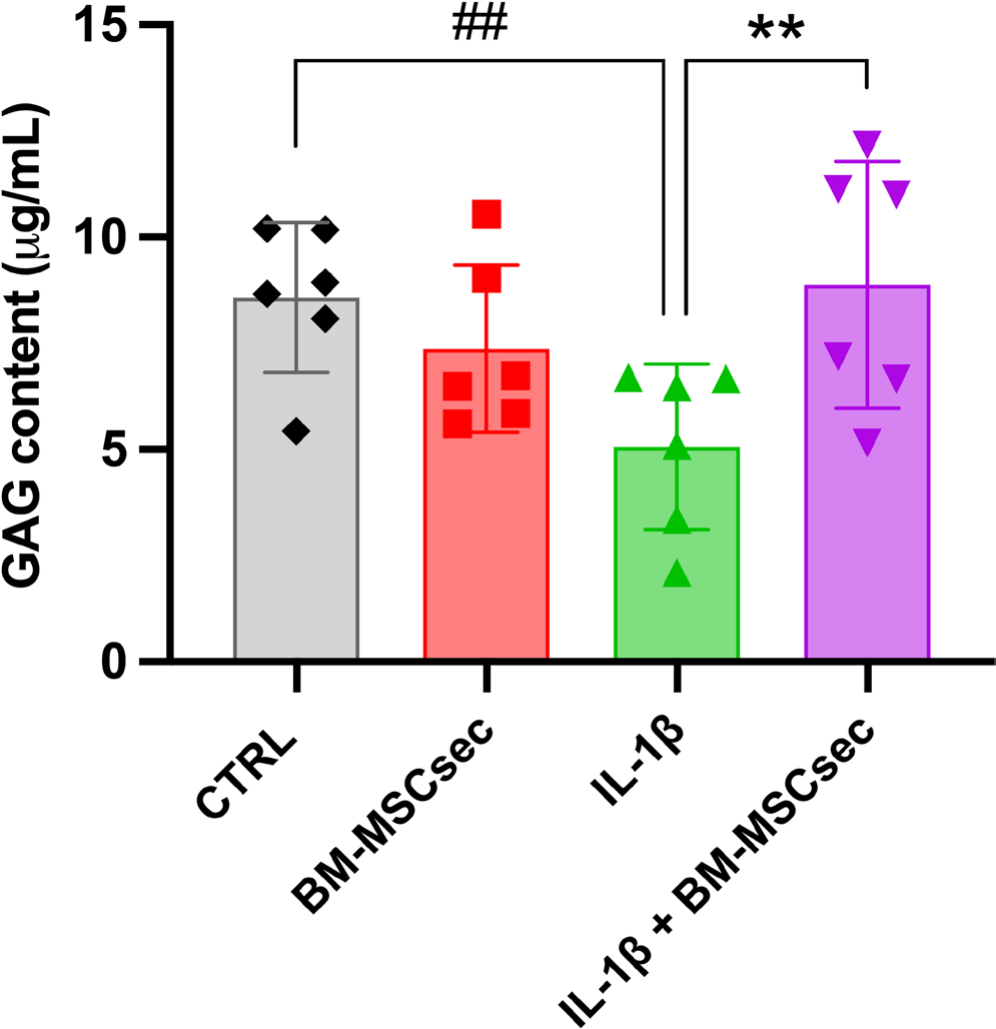
BM‐MSCsec enhanced GAG synthesis in IL‐1β‐pretreated hNPCs. hNPCs treated with IL‐1β and BM‐MSCsec showed significantly higher GAG levels compared to cells treated with IL‐1β alone (*n* = 6). Data were analyzed through one‐way ANOVA with Dunnett's multiple comparisons test where each group was compared with IL‐1β. **p < 0.01. compared to the control group; ^##^ *p* < 0.01 compared to the IL‐1β group. Abbreviations: BM‐MSCsec = bone marrow–derived mesenchymal stromal cell secretome; GAG = glycosaminoglycan; hNPCs = human nucleus pulposus cells; IL = interleukin.

hNPCs embedded in alginate beads (*n* = 6) and exposed to IL‐1β showed a significant reduction in GAG compared with naïve controls (5.06 ± 1.95 vs. 8.58 ± 1.76 μg/mL, *p* = 0.0095). Co‐treatment with BM‐MSCsec significantly counteracted the IL‐1β‐induced decrease in GAG content (8.87 ± 2.90 μg/mL, *p* = 0.0070) (Figure 5), preserving GAG levels toward those observed in the naïve control group compared with IL‐1β alone (8.87 ± 2.90 μg/mL, *p* = 0.0070; Figure 5).

**FIGURE 6:**
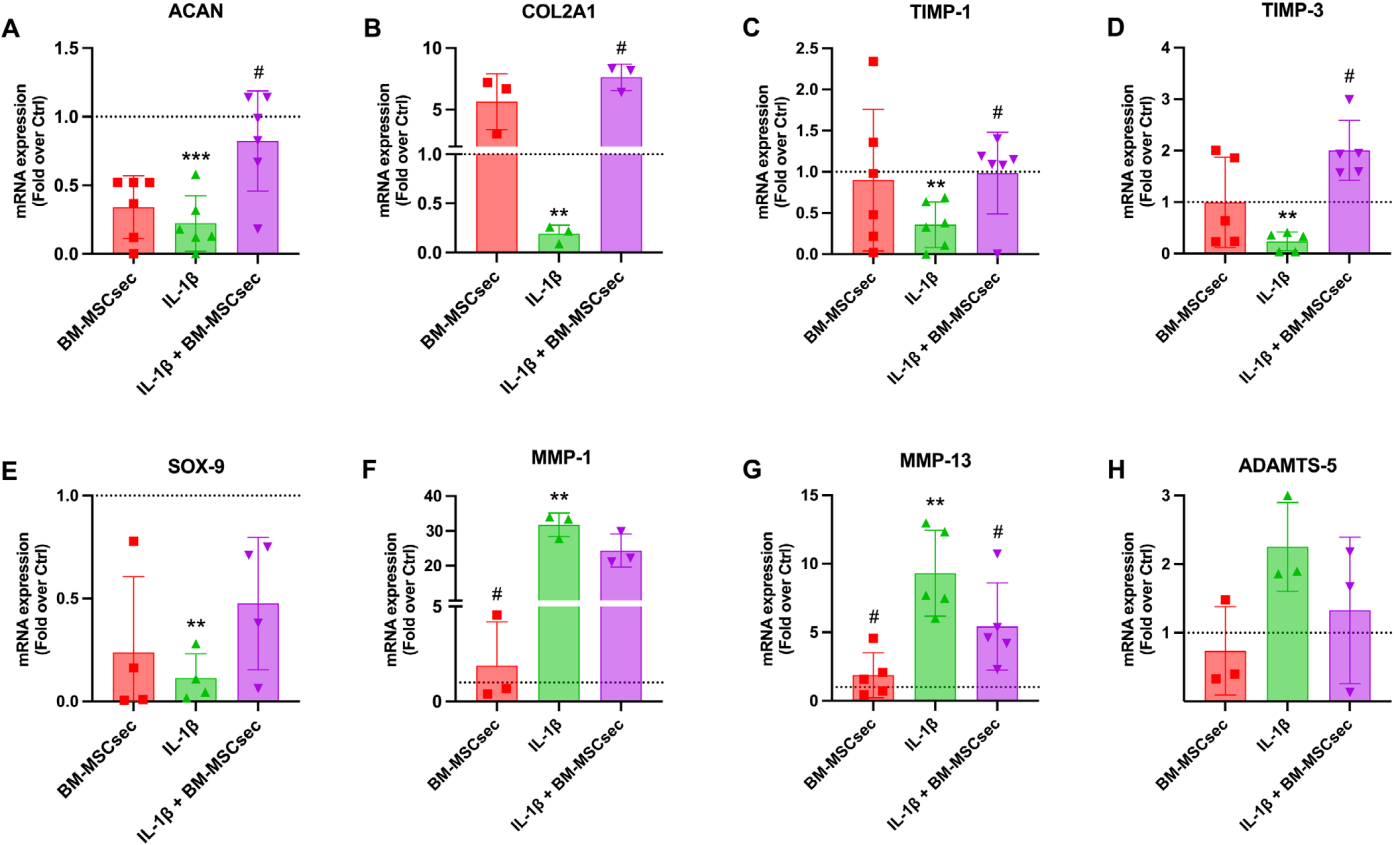
BM‐MSCsec promoted the expression of ECM markers and suppressed catabolic gene expression in hNPCs. After 7 days of treatment, hNPCs exposed to IL‐1β + BM‐MSCsec showed significantly increased expression of *ACAN* (A), *COL2A1* (B), *TIMP‐1* (C), *TIMP‐3* (D), and *SOX‐9* (E), while expression levels of the catabolic genes *MMP‐1* (F) and *MMP‐13* (G) were significantly reduced compared to IL‐1β alone, which significantly decreased anabolic gene expression (A–E) and upregulated catabolic genes (F, G). No significant differences were found regarding *ADAMTS‐5* expression (H). Data were analyzed through one‐way ANOVA with Dunnett's multiple comparisons tests where each group was compared with IL‐1β. See the Results section for details on sample sizes. Data were normalized based on *GAPDH* and *actin* expression and calculated as fold change compared to the controls. The dashed line indicates the reference value (y = 1), corresponding to the baseline for fold change normalization. **p < 0.01, ***p < 0.001 compared to the control group; ^#^ *p* < 0.05 compared to the IL‐1β group. Abbreviations: ACAN = aggrecan; ADAMTS = a disintegrin and metalloproteinase with thrombospondin motifs; BM‐MSCsec = bone marrow–derived mesenchymal stromal cell secretome; COL2A1 = collagen type II; hNPCs = human nucleus pulposus cells; IL = interleukin; MMP = matrix metalloproteinase; SOX = SRY‐Box Transcription Factor; TIMP = Tissue Inhibitors of Metalloproteinase Inhibitors.

After 7 days, IL‐1β‐primed BM‐MSCsec partially mitigated the IL‐1β‐induced suppression of anabolic and matrix‐regulatory gene expression in hNPCs. Specifically, co‐treatment with BM‐MSCsec significantly reduced the decrease in *ACAN* expression observed after IL‐1β stimulation, although expression remained lower than in untreated controls (0.82 ± 0.136, *p* = 0.0150, *n* = 6; Figure 6A). Compared with IL‐1β alone, co‐treatment also resulted in significantly higher expression of *COL2A1* (7.63 ± 1.07, *p* = 0.0110, *n* = 3; Figure 6B), *TIMP‐1* (0.99 ± 0.50, *p* = 0.0379, *n* = 6; Figure 6C), and *TIMP‐3* (2.01 ± 0.58, *p* = 0.0125, *n* = 5; Figure 6D), supporting a protective effect of BM‐MSCsec on ECM‐related gene expression in an inflammatory microenvironment.

In contrast, IL‐1β alone significantly downregulated these genes (*ACAN*: 0.22 ± 0.20, *p* = 0.0006; *COL2A1*: 0.19 ± 0.09, *p* = 0.0077; *TIMP‐1*: 0.36 ± 0.28, *p* = 0.0056; *TIMP‐3*: 0.24 ± 0.18, *p* = 0.0017), including *SOX‐9* (0.11 ± 0.12, *p* = 0.0014, *n* = 4; Figure 6E), while increasing the expression of *MMP‐1* (31.76 ± 3.42, *p* = 0.0079, *n* = 3; Figure 6F) and *MMP‐13* (9.31 ± 3.14, *p* = 0.0093, *n* = 5; Figure 6G) compared to controls. Under basal conditions, BM‐MSCsec alone induced a modest increase in *MMP‐1* expression compared with untreated controls (1.88 ± 2.31, *p* = 0.0230); however, this increase was significantly lower than that observed following IL‐1β stimulation. Similarly, *MMP‐13* expression in BM‐MSCsec‐treated hNPCs remained significantly lower than in IL‐1β‐treated cells (1.88 ± 1.64, *p* = 0.0424), indicating that IL‐1β elicited a markedly stronger catabolic response than BM‐MSCsec alone. Conversely, no significant differences were found regarding *ADAMTS‐5* expression (*n* = 3) among all groups (Figure 6H).

### 3.5 IL‐1β‐Primed BM‐MSCsec Blunted Inflammatory Gene Expression in hNPCs

**FIGURE 7:**
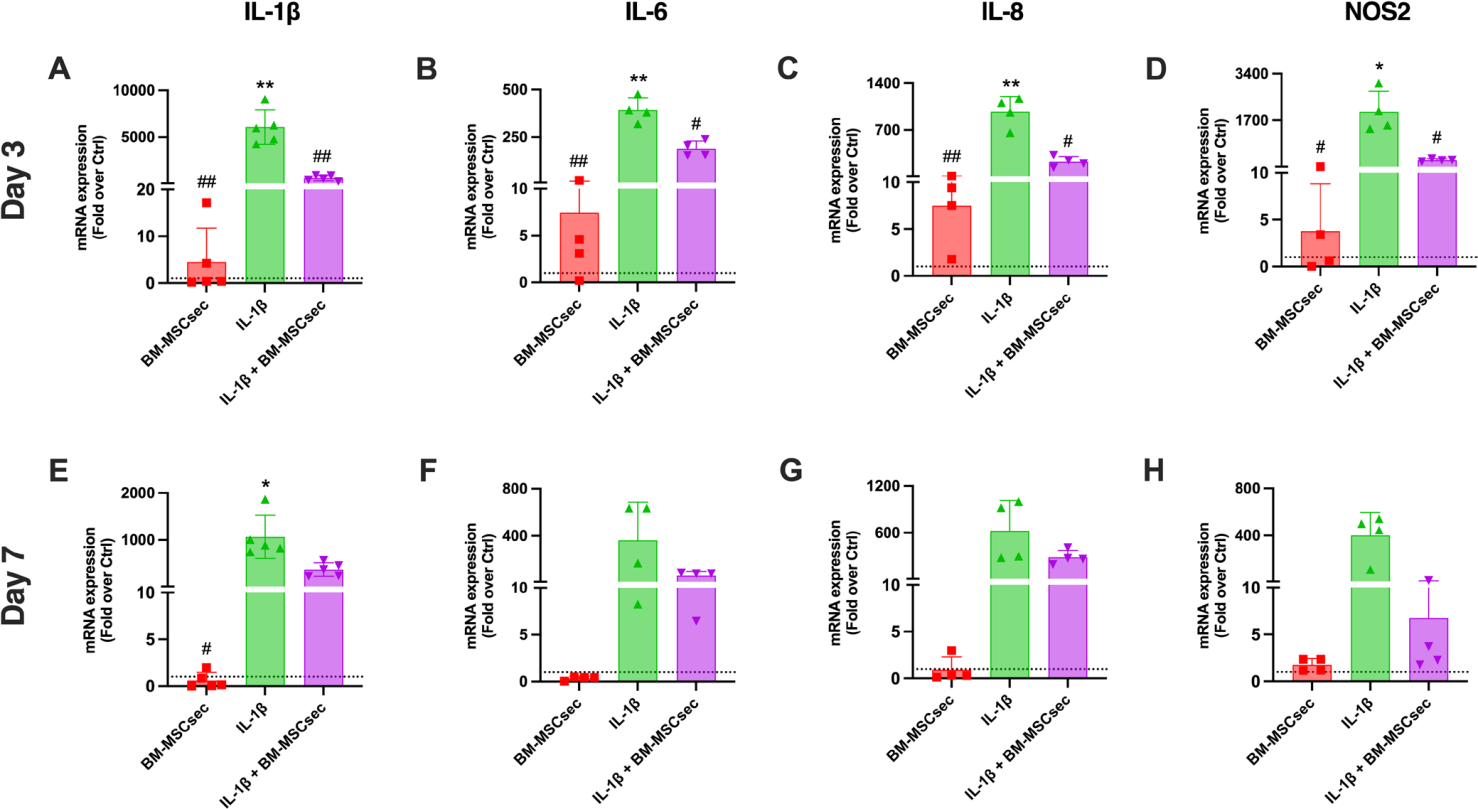
IL‐1β–primed BM‐MSCsec attenuated the inflammatory gene expression profile of hNPCs. Treatment with BM‐MSCsec led to a reduction in mRNA levels of *IL‐1β* (A), *IL‐6 (*B), *IL‐8* (C), and *NOS2* (D) at day 3 compared to the IL‐1β group, with this anti‐inflammatory trend persisting through day 7 (E–H). Data were analyzed through one‐way ANOVA with Dunnett's multiple comparisons tests where each group was compared with IL‐1β. See the Results section for details on sample sizes. Data were normalized based on *GAPDH* and *actin* expression and calculated as fold change compared to the controls. The dashed line indicates the reference value (*y* = 1), corresponding to the baseline for fold change normalization. *p < 0.05, **p < 0.01, compared to the control group; ^#^ *p* < 0.05, ^##^ *p* < 0.01 compared to the IL‐1β group. Abbreviations: BM‐MSCsec = bone marrow–derived mesenchymal stromal cell secretome; hNPCs = human nucleus pulposus cells; IL = interleukin; NOS = nitric oxide synthase.

At day 3, hNPCs treated with IL‐1β + BM‐MSCsec exhibited a significant decrease of *IL‐1β* (6066.0 ± 1855.0 vs. 604.2 ± 282.8, *p* = 0.0061, *n* = 5; Figure 7A), *IL‐6* (372.1 ± 354.3 vs. 3064.0 ± 233.6, *p* = 0.0035, *n* = 4; Figure 7B), *IL‐8* (225.4 ± 78.2 vs. 975.0 ± 226.3, *p* = 0.0072, *n* = 4; Figure 7C), and *NOS2* expression (249.5 ± 45.2 vs. 2007.0 ± 755.8, *p* = 0.0043, *n* = 4; Figure 7D) compared to the IL‐1β group. Treatment with IL‐1β alone resulted in a statistically significant increase in these inflammatory markers versus the control group (*p* < 0.05 for *NOS2*; *p* < 0.001 for *IL‐1β, IL‐6* and *IL‐8*). At day 7, except for *IL‐1β* (0.6 ± 0.8 in the BM‐MSCsec group vs. 1067.0 ± 457.3 in the IL‐1β group, *p* = 0.0105; Figure 7E), BM‐MSCsec did not produce a significant effect, although the trend in inflammatory marker expression was similar to that observed at day 3 (Figure 7E–H; *n* = 5 for *IL‐1β*; *n* = 4 for *IL‐6, IL‐8* and *NOS2*). These results suggest that BM‐MSCsec can transiently attenuate IL‐1β‐induced inflammatory gene expression in hNPCs, with the most pronounced effects observed at an early time point.

### 3.6 BM‐MSCsec Alleviated hNPCs Senescence

**FIGURE 8:**
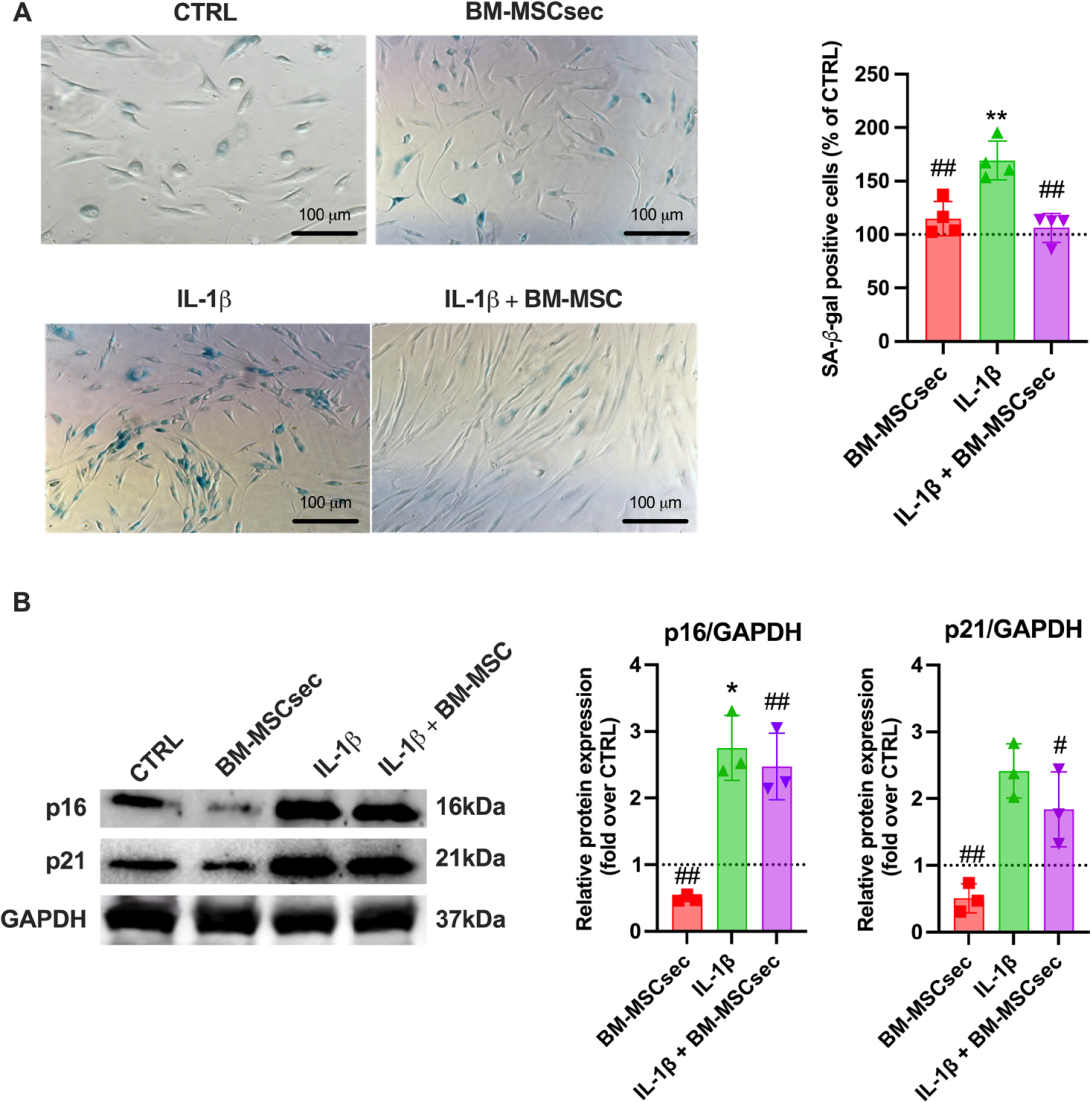
BM‐MSCsec blunted hNPCs senescence. (A) Representative images of SA‐β‐gal–stained hNPCs showing a significant reduction in the percentage of senescent cells following treatment with BM‐MSCsec, compared to cells cultured with IL‐1β alone (*n* = 4). The dashed line indicates a reference value of y = 100 (%). Blue staining indicates senescent cells. 20 × magnification. Scale bars = 100 μm. (B) Western blot analysis of senescence markers p16 and p21 (*n* = 4). BM‐MSCsec treatment reduced protein expression of both markers under basal conditions and in hNPCs pretreated with IL‐1β. Results are expressed as fold changes over the control. The dashed line indicates the reference value (*y* = 1), corresponding to the baseline for fold change normalization. Data were analyzed through one‐way ANOVA with Dunnett's multiple comparisons tests where each group was compared with IL‐1β. *p < 0.05, **p < 0.01 compared to the control group; ^#^ *p* < 0.05, ^##^p < 0.01 compared to the IL‐1β group. Abbreviations: BM‐MSCsec = bone marrow–derived mesenchymal stromal cell secretome; hNPCs = human nucleus pulposus cells; IL = interleukin; SA‐β‐gal = senescence‐associated β‐galactosidase.

SA‐β‐gal staining revealed that IL‐1β‐preconditioned BM‐MSCsec treatment significantly reduced senescence in hNPCs (*n* = 4) compared to cells exposed to IL‐1β (17.9% ± 1.6% and 16.3% ± 1.3% vs. 26.5% ± 3.1%, *p* = 0.0068 and 0.0092; Figure 8A). On the other hand, IL‐1β alone promoted a pro‐senescent effect relative to the control group (15.8% ± 2.5%, *p* = 0.0038). Western blot further confirmed that both BM‐MSCsec alone and IL‐1β‐primed BM‐MSCsec treatment lowered the protein levels of p16 (0.5 ± 0.1 and 2.5 ± 0.5, *p* < 0.01) and p21 (0.5 ± 0.2 and 1.8 ± 0.6, *p* < 0.05) in hNPCs (*n* = 4) compared to IL‐1β (2.8 ± 0.5 and 2.4 ± 0.4, respectively; Figure 8B). These reductions were more pronounced than those observed in hNPCs treated with IL‐1β only (*p* < 0.05 vs. control).

## 4 Discussion

In this study, the secretome derived from IL‐1β‐primed BM‐MSCs partially counteracted the detrimental effects of IL‐1β on hNPCs, supporting cell proliferation and viability, reducing oxidative stress, preserving GAG deposition and ECM‐related gene expression, downregulating inflammatory mediators, and attenuating cell senescence compared with IL‐1β‐treated hNPCs. These effects were particularly pronounced when BM‐MSCsec was administered to hNPCs previously exposed to IL‐1β, highlighting its potential to counteract inflammation‐induced degenerative changes in vitro.

IDD is an aging‐related condition characterized by the early loss of NPC progenitors, increased expression of catabolic mediators, and progressive breakdown of the NP matrix [23, 24, 25]. These pathological changes lead to progressive cell death, disc height loss, and alterations of the disc's mechanical properties [26]. Cell‐based treatments, typically involving the intradiscal injection of healthy cells isolated from various sources and expanded in vitro, aim to slow or reverse IDD progression through cellular repopulation and immunomodulation [27, 28]. Although preclinical studies have demonstrated promising biological effects of cell therapies, numerous clinical trials have yielded limited or inconclusive results [29]. This inconsistency hampers the evaluation of therapeutic outcomes and limits the translation of findings into clinical practice [30, 31].

Emerging evidence suggests that the putative therapeutic effects of intradiscal cell therapy may be mediated largely by paracrine mechanisms. Rather than relying solely on direct engraftment and differentiation at the injection site, transplanted cells release a range of bioactive molecules that modulate the local microenvironment and influence the behaviour of surrounding cells. These trophic factors hold a fundamental role in orchestrating complex biological processes implicated in disc repair and regeneration [32]. Therefore, various priming approaches have recently been investigated to enhance MSC therapeutic effects by boosting their paracrine activity [33]. IDD is intrinsically characterized by high levels of proinflammatory cytokines and associated chemokines, which not only facilitate immune cell recruitment but also upregulate catabolic enzymes such as MMPs and aggrecanases, ultimately perpetuating a cycle of inflammation and degeneration [34, 35, 36]. Consistent with previous findings, our prior study confirmed that the BM‐MSCsec significantly enhanced GAG production by upregulating *ACAN* and *SOX‐9* gene expression while simultaneously reducing *MMP‐13*, *IL‐6*, and *ADAMTS‐5* expression in IL‐1β‐inflamed hNPCs [6]. Gonçalves et al. [37] examined the effects of the MSC secretome on AF cells subjected to IL‐1β stimulation and mechanical loading. While IL‐1β strongly increased the expression of catabolic markers and accelerated ECM breakdown, the MSC secretome exerted an anti‐proteolytic effect by downregulating *MMP‐1*, *MMP‐2*, *MMP‐3*, and *MMP‐9* expression.

The early increase in proliferation observed in IL‐1β‐treated hNPCs suggests a significant reparative response to inflammatory injury, which has already been described in other studies [6, 38, 39]. Interestingly, the addition of preconditioned BM‐MSCsec attenuated this IL‐1β‐induced proliferative upregulation, resulting in a modest increase in cell number. This modulation may reflect a regulatory effect of the secretome on the aberrant activation state induced by inflammation. Moreover, the reduced cell death observed in hNPCs following treatment with IL‐1β and BM‐MSCsec suggests a functional interaction between secreted bioactive factors, including cytokines, growth factors, and EVs, and the recipient cells. The loss of viable NP progenitor cells critically impairs the disc's intrinsic regenerative capacity, thereby accelerating IDD progression [40]. Our findings indicate that BM‐MSCsec may preserve cell viability, especially when hNPCs are already exposed to an inflammatory microenvironment.

Another key barrier to effective cell therapy is oxidative stress, which promotes cell senescence, apoptosis, and impaired differentiation. These detrimental effects compromise both the survival and regenerative capacity of transplanted cells, thereby limiting their therapeutic efficacy [41]. In our study, we demonstrated that BM‐MSCsec attenuated nitrite‐induced oxidative stress and reduced intracellular ROS levels by modulating cell antioxidant mechanisms and exerting anti‐inflammatory effects. The MSC secretome is highly dynamic and responsive to the surrounding microenvironment, especially in the presence of catabolic stimuli. When exposed to proinflammatory cytokines such as IFN‐γ, TNF‐α, or IL‐1β, MSCs undergo a shift in their secretory profile, leading to the release of key immunomodulatory factors such as prostaglandin E2, indoleamine 2,3‐dioxygenase, IL‐10, and NO. These paracrine mediators, along with cell–cell interactions, contribute to the immunomodulatory effects of MSCs, such as suppression of T‐cell proliferation, modulation of natural killer cell activity, and a shift in cytokine balance toward an anti‐inflammatory profile [11, 42]. Wangler et al. [43] investigated how the MSC secretome varied in response to different disc microenvironments, namely healthy, traumatic, or degenerative. Interestingly, MSCs exposed to NPCs derived from traumatic or degenerative discs showed marked alterations in their secretome composition, with a higher content of proteins related to inflammation and tissue repair, thereby highlighting the MSCs' adaptive response to injury and degeneration. Similarly, Ferreira and colleagues [44] showed that the secretome extracted from IL‐1β‐preconditioned MSCs modulated the inflammatory response and promoted aggrecan synthesis in an ex vivo model of IDD.

The detailed mechanisms underlying MSC secretory activity remain elusive. In our study, the secretome derived from IL‐1β‐preconditioned human BM‐MSCs, intended to mimic the inflammatory conditions of degenerative IVD, effectively downregulated the expression of key proinflammatory cytokines (IL‐1β, IL‐6, IL‐8, and NOS2). We postulate that the observed short‐term effects may result from a decreased responsiveness of NPCs over time or from the acute nature of the MSC priming, which involved a brief, high‐dose IL‐1β stimulation prior to secretome collection. Indeed, while several preconditioning strategies have been described in the literature [11, 45, 46], methodological parameters such as the passage number of MSCs, duration of stimulation, secretome concentration, and collection protocols are often poorly reported and remain a critical area lacking standardization. Intriguingly, our study also provides insights into the potential of the MSC secretome to attenuate senescence in NPCs. Senescence in disc cells is a hallmark of aging and degeneration, characterized by the acquisition of a senescence‐associated secretory phenotype, which is influenced by both intrinsic and extrinsic factors. Although the exact regulatory mechanisms are still under investigation, the detrimental role of senescent cells in IDD progression has been well documented in both in vitro and in vivo models [47, 48]. Various stimuli, such as oxidative stress, inflammatory cytokines, and genotoxic insults, are known to induce the expression of cell cycle inhibitors including p21, p53, and p16INK4a, resulting in irreversible growth arrest [49]. Using SA‐β‐gal staining as well as immunodetection of p21 and p16INK4a, we showed that the BM‐MSCsec significantly alleviated IL‐1β‐induced senescence in hNPCs. This anti‐senescent effect may represent an important therapeutic mechanism of the MSC‐derived secretome. Further supporting the paracrine impact of senescent cells in IDD, a recent study demonstrated that TNF‐α‐induced senescent NPCs, when co‐cultured with inner AF cells, promoted degenerative changes in the AF cell population. This highlights a mechanism whereby the secretome of senescent NPCs can propagate senescence and degeneration to neighbouring disc cells [50].

Finally, the use of the MSC secretome presents a promising and more accessible alternative to traditional cell‐based treatments in regenerative medicine. One major advantage of secretome‐based approaches is the reduced risk of immunogenicity, eliminating the need for donor–recipient matching required in cell transplantation. As a cell‐free product, MSC‐conditioned medium bypasses the strict regulatory and handling requirements associated with live cell administration, making it easier to use in clinical settings. Secretome preparation also avoids the time‐consuming expansion required for cell therapy, improving cost‐efficiency and scalability. Additionally, the secretome can be stored in frozen or lyophilized form, simplifying transportation and removing the need for cryopreservation [51]. However, accurate characterization of the MSC secretome is essential, as its therapeutic potential is influenced by several factors, including the MSC source, donor age, preconditioning protocols, and the composition of the culture medium. At present, several challenges remain unresolved, such as the lack of good manufacturing product‐compliant production methods, standardized storage procedures, shelf‐life data, and quality control criteria, each of which is critical to ensure safety, consistency, and clinical efficacy. Further research is also needed to determine optimal dosing, frequency of administration, and injection volume to maximize therapeutic outcomes [52]. It is worth noting that the MSC secretome is composed of both soluble and vesicular components, with EVs increasingly recognized as the principal mediators of its regenerative effects [53]. Recently, Ambrosio et al. [38] highlighted the importance of EV source, showing that NPC‐derived EVs may offer a more effective therapeutic approach for treating IDD and associated pain compared to the more commonly used MSC‐derived EVs.

Despite these promising findings, this study has several limitations. While in vitro experimental models provide valuable mechanistic insights, they represent a simplified approximation of the native tissue environment. Alginate bead cultures were employed to preserve a three‐dimensional microenvironment and support a disc‐like NP cell phenotype; however, they do not fully recapitulate the complex biomechanical, nutritional, and biochemical gradients of the native IVD, including dynamic mechanical loading, hypoxia, and interactions with ECM components. In contrast, monolayer cultures allow high‐resolution analysis of cell morphology, intracellular signaling, and viability‐related parameters, but are associated with well‐recognized limitations such as phenotypic drift, loss of three‐dimensional cell–cell and cell–matrix interactions, and altered gene expression profiles compared with the native NP tissue. Accordingly, findings derived from monolayer cultures should be interpreted primarily as mechanistic observations rather than direct predictors of in vivo cellular behaviour. Furthermore, the hNPCs used were derived from patients who were not stratified by the severity or anatomical level of IDD. Donor variability, due to factors including age, sex, and comorbidities, may have influenced the cellular responses. The BMSC secretome was not subjected to proteomic or bioinformatic analysis, limiting insights into its actual composition. Future studies should aim to identify and characterize the molecular profile of MSC‐derived secretome under different pathological disc conditions to better understand the mechanisms underlying its therapeutic effects. Such investigations may enable the development of more targeted, cell‐free strategies for enhancing disc repair and regeneration. Additionally, because the secretome was administered as a 1:1 dilution with standard culture medium, the possibility that some effects were influenced by reduced nutrient availability in the BM‐MSCsec group cannot be entirely excluded. This dilution strategy was intentionally adopted to better isolate the biological activity of secretome‐derived factors, thereby attributing observed effects more directly to its composition rather than to differences in nutrient content. Importantly, FBS, glutamine, and P/S were supplemented at identical final concentrations across both CTRL and BM‐MSCsec conditions to ensure comparable baseline nutrient support. The secretome itself consisted of serum‐free conditioned medium, collected in the absence of FBS to minimize variability and avoid confounding effects from exogenous serum‐derived components.

This study demonstrated that IL‐1β‐preconditioned MSC secretome protects hNPCs from inflammation‐driven IDD by modulating proliferation, oxidative stress, ECM homeostasis, inflammatory signaling, and cellular senescence. By harnessing and standardizing this paracrine‐mediated approach, future cell‐free therapies could overcome key limitations of cell transplantation, possibly offering a scalable, off‐the‐shelf solution for IDD. Further molecular dissection of the secretome and in vivo validation will be critical to advance these findings toward clinical application.

## Author Contributions

**Veronica Tilotta:** conceptualization, methodology, investigation, writing – original draft. **Claudia Cicione:** investigation, methodology. **Giuseppina Di Giacomo:** methodology, investigation, data curation. **Rocco Papalia:** supervision, visualization, writing – review and editing. **Luca Ambrosio:** data curation, formal analysis, writing – review and editing, validation. **Vincenzo Denaro:** visualization, writing – review and editing, supervision. **Fabrizio Russo:** visualization, writing – review and editing, supervision. **Gianluca Vadalà:** funding acquisition, investigation, supervision, visualization, conceptualization.

## Funding

This work was supported by NextGenerationEU, NRRP PNC‐E3‐2022‐23683269 PNC‐HLS‐TA (CUP code: F83C22002880001).

## Conflicts of Interest

Gianluca Vadalà is an Editorial Board member of JOR Spine and a co‐author of this article. To minimize bias, he was excluded from all editorial decision‐making related to the acceptance of this article for publication. The other authors declare no conflicts of interest.

## Data Availability

The data that support the findings of this study are available from the corresponding author upon reasonable request.
